# Clinical Potential of an Enzyme-based Novel Therapy for Cocaine Overdose

**DOI:** 10.1038/s41598-017-14105-5

**Published:** 2017-11-10

**Authors:** Ting Zhang, Xirong Zheng, Ziyuan Zhou, Xiabin Chen, Zhenyu Jin, Jing Deng, Chang-Guo Zhan, Fang Zheng

**Affiliations:** 1 Molecular Modeling and Biopharmaceutical Center, College of Pharmacy, University of Kentucky, 789 South Limestone Street, Lexington, KY 40536 USA; 2 Department of Pharmaceutical Sciences, College of Pharmacy, University of Kentucky, 789 South Limestone Street, Lexington, KY 40536 USA

## Abstract

It is a grand challenge to develop a truly effective medication for treatment of cocaine overdose. The current available, practical emergence treatment for cocaine overdose includes administration of a benzodiazepine anticonvulsant agent (*e.g*. diazepam) and/or physical cooling with an aim to relieve the symptoms. The inherent difficulties of antagonizing physiological effects of drugs in the central nervous system have led to exploring protein-based pharmacokinetic approaches using biologics like vaccines, monoclonal antibodies, and enzymes. However, none of the pharmacokinetic agents has demonstrated convincing preclinical evidence of clinical potential for drug overdose treatment without a question mark on the timing used in the animal models. Here we report the use of animal models, including locomotor activity, protection, and rescue experiments in rats, of drug toxicity treatment with clinically relevant timing for the first time. It has been demonstrated that an efficient cocaine-metabolizing enzyme developed in our previous studies can rapidly reverse the cocaine toxicity whenever the enzyme is given to a living rat, demonstrating promising clinical potential of an enzyme-based novel therapy for cocaine overdose as a successful example in comparison with the commonly used diazepam.

## Introduction

Development of a truly effective medication for treatment of drug abuse is a grand challenge^1,2^. As well known, a truly effective medication for drug abuse must be able to effectively block/reverse physiological/toxic effects of the drug and prevent relapse during abstinence without affecting normal functions of the brain. The currently available pharmacological approaches to drug abuse treatment either affect normal functions of some brain receptors/transporters or are unable to prevent relapse. It is highly desired to develop a better therapeutic strategy which can effectively block/reverse physiological/toxic effects of the drug and prevent relapse without altering normal functions of the neural circuits. Cocaine abuse is a compelling example of how traditional pharmacodynamic approach using an agonist or antagonist is difficult to work^3^. Despite decades of efforts, none of pharmacodynamic agents tested so far has been proven effective for treatment of cocaine addiction or overdose. There is still no FDA-approved medication specific for cocaine addiction or overdose.

The toxicity induced by cocaine in animals and humans is attributed to its multiple physiological effects in central nervous system and cardiovascular system, among others^4^. Like other abused drugs, cocaine use leads to neurological impairments due to its neurotoxic effects mediated by several dopaminergic and glutamatergic neurotransmitter systems^5,6^. In severe cases, the acute toxicity associated with cocaine overdose could cause life-threatening events including seizures, cardiovascular failure, or respiratory depression^4^. Practical emergence treatment for cocaine intoxication includes initial administration of a benzodiazepine anticonvulsant agent (*e.g*. diazepam) and/or physical cooling, followed by interventions aimed to relieve the other symptoms^7^. The inherent difficulties of antagonizing physiological effects of drugs in the central nervous system have led to exploring protein-based pharmacokinetic approaches using biologics like vaccines, monoclonal antibodies, and enzymes^3,8^. However, none of the pharmacokinetic agents has demonstrated convincing preclinical evidence of clinical potential for drug overdose treatment without a question mark on the timing^9^ used in the animal models. Here we report the use of animal models of drug toxicity treatment with clinically relevant timing for the first time, demonstrating promising clinical potential of an enzyme-based novel therapy for cocaine overdose as a successful example in comparison with the commonly used diazepam.

In principle, of various pharmacokinetic approaches, an efficient drug-metabolizing enzyme should be much more efficient for drug addiction and overdose treatment^3,10–12^. In particular, each enzyme molecule can degrade multiple drug molecules, depending on the catalytic parameters of the enzyme such as the turnover number (catalytic rate constant *k*_cat_), remarkably different from the well-known stoichiometric binding of an antibody with drug. Based on our structure-and-mechanism-based computational design, efficient and thermally stable cocaine-metabolizing enzymes have been discovered and developed recently as potential candidates of therapies for cocaine overdose and addiction^13–18^. These computationally designed enzymes, that are mutants of human butyrylcholinesterase (BChE) or bacterial cocaine esterase (CocE), can rapidly convert cocaine to physiologically inactive ecgonine methyl ester (EME) and benzoic acid. In particular, our designed human BChE mutants are recognized as *true* cocaine hydrolases (CocHs), with at least 1,000-fold improved catalytic efficiency against (-)-cocaine compared to wild-type human BChE^13–16^. The first one of our designed CocHs, known as CocH1 (the A199S/S287G/A328W/Y332G mutant of human BChE)^13,19^, truncated after amino acid #529, was fused with human serum albumin (HSA) to prolong the biological half-life^12^. This HSA-fused BChE mutant is also known as Albu-CocH, Albu-CocH1, AlbuBChE, or TV-1380 in the literature^10–12,20^. TV-1380 has been proven safe and effective for use in humans to accelerate cocaine metabolism in Phase I clinical trials for cocaine addiction treatment^10,11^, but its therapeutic value for cocaine overdose treatment has not been explored in any clinical trial, without convincing preclinical data to demonstrate the clinical potential for cocaine overdose treatment.

Preclinical studies on Albu-CocH1 and other mutants of BChE or CocE for cocaine toxicity treatment in mice or rats reported so far included the protection and rescue experiments. In the protection experiment, the animals were pretreated intravenously (i.v.) with an enzyme before intraperitoneal (i.p.) injection of a lethal dose of cocaine (*e.g*. 180 mg/kg, LD_100_)^14,15,17,21–23^. In the rescue experiment, the enzyme was administered right after the onset of cocaine-induced convulsion (which usually occurred within first three minutes after i.p. injection of a lethal dose of cocaine)^12,24,25^. The timing used in these animal models raised concerns about the clinical irrelevance; it was regarded as “a difficult transition to the bedside”^9^. In fact, more than 40% of patients present to the emergency department with cocaine toxicity more than 1 hour after cocaine use^9^. The protection and rescue experiments reported so far have no indication concerning whether the enzyme therapy will be effective in these late presenters.

For an appropriate use of animal models with clinically relevant timing, one first needs to understand the general features of pharmacokinetic (PK) profiles of cocaine in the body. It should be noted that the elimination half-life of cocaine in the blood is dependent on the dose used in the PK study^26,27^, because all endogenous cocaine-metabolizing enzymes are saturated when cocaine concentration is sufficiently high in plasma. A pharmacological dose (50 mg or ~1 mg/kg) of cocaine had an average elimination half-life of ~93 min in humans, and significant blood cocaine concentrations were detected at 12 hours after cocaine infusion^28^. Significantly higher doses of cocaine under the overdose conditions are expected to have longer elimination half-lives and physiological/toxic effects for a much longer period of time in humans. With the dose-dependence of cocaine elimination half-life in mind, it is not very difficult to understand why many patients suffered acute myocardial infarction at an average of 18 hours after cocaine use^29^. In cocaine overdose caused deaths, the cocaine concentrations at the time of death are difficult to determine because plasma enzymes, including BChE, in the blood metabolize cocaine^27^ such that cocaine levels in the blood can continuously drop after death and even in the lab tube after blood is drawn^30^. As a result, in an autopsy study of 37 patients^31^, the determined blood cocaine concentrations ranged from 31 to 0.04 mg/L (or from 102 to 0.13 µM) when the autopsy was carried out several hours to days after death.

Rats metabolize cocaine in the same pathways as humans, although rats can eliminate cocaine more rapidly than humans. Thus, cocaine generally has a significantly shorter elimination half-life in rats compared to humans. For example, 5 mg/kg cocaine (administered i.v.) had an elimination half-life of only ~23 min and did not show significant concentration of cocaine after 60 min following the cocaine administration according to our previously reported study^32^. So, a period of 30 min for cocaine detoxification in rats might be corresponding to a period of quite a few hours for cocaine detoxification in humans. We aimed to know whether the enzyme Albu-CocH1 can be effective in cocaine toxicity treatment when Albu-CocH1 is administered at 30 min in rats (equivalent to a few hours in humans) after the cocaine administration using animal behavior studies combined with blood sample analyses.

## Results

### Cocaine PK profile and cocaine-induced hyperactivity

**Figure 1:**
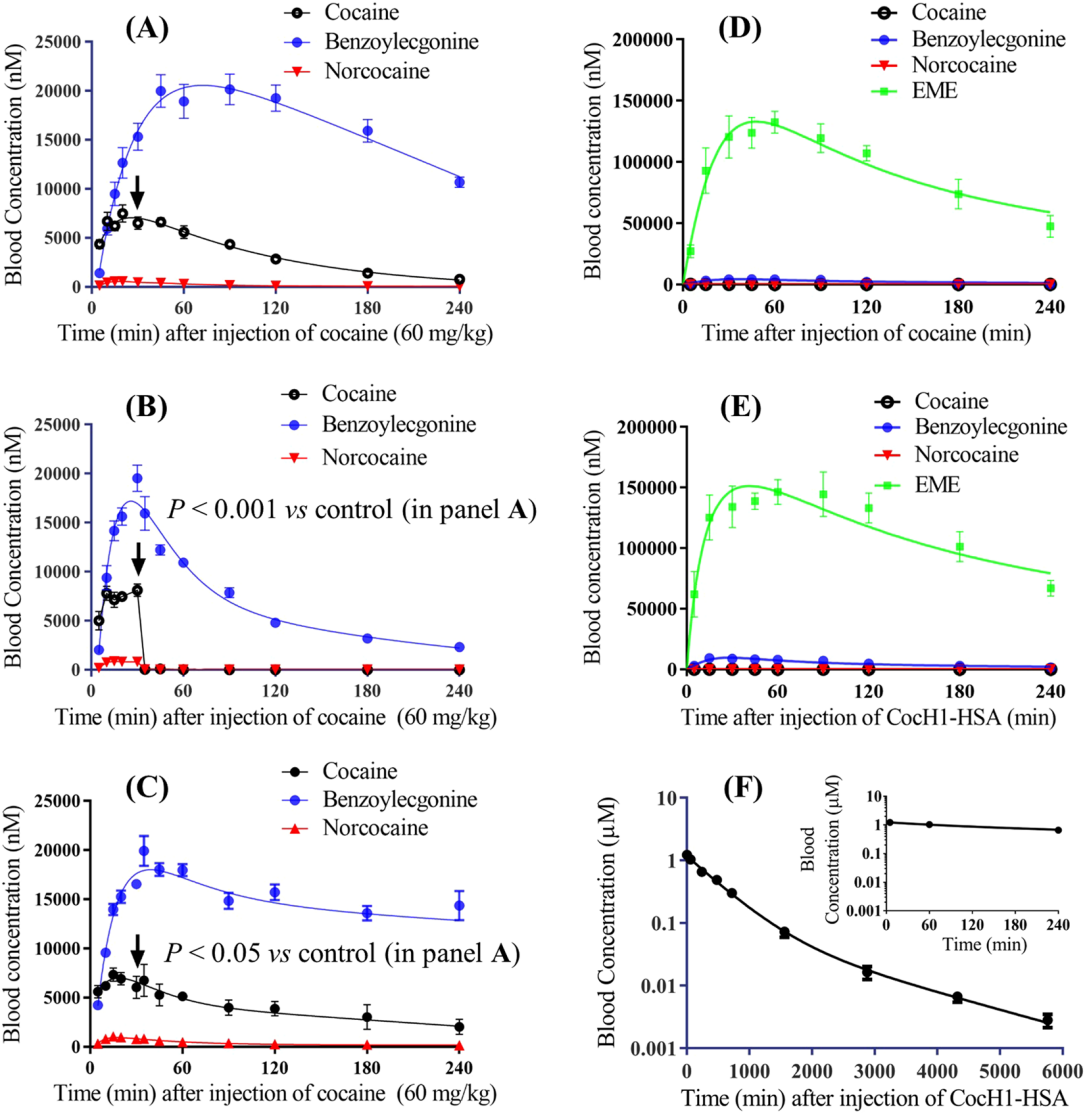
Time-dependent blood concentrations of cocaine, cocaine metabolites, and Albu-CocH1 in rats (n = 5 for each group). (**A**–**C**) Cocaine pharmacokinetics after i.p. administration of 60 mg/kg cocaine without (**A**) and with i.v. administration of 5 mg/kg Albu-CocH1 (**B**) or i.p. administration of 60 mg/kg cocaine (**C**) at *t* = 30 min. Black arrows indicate the time point when the intervention was introduced. (**D**) Cocaine pharmacokinetics after i.p. administration of 180 mg/kg cocaine in rats pretreated with 5 mg/kg Albu-CocH1 (i.v.) 1 min before the cocaine administration (protection experiment). (**E**) Cocaine pharmacokinetics after the Albu-CocH1 administration (*t* = 0) in rescue experiment – rats were first given 180 mg/kg cocaine (i.p.) and then 5 mg/kg Albu-CocH1 (i.v.) in 1 min after the onset of cocaine-induced convulsion; blood samples were taken only after the Albu-CocH1 administration. (**F**) Albu-CocH1 pharmacokinetics after i.v. administration of 5 mg/kg Albu-CocH1. *p* < 0.05 indicates the significant differences in the pharmacokinetic data between the treated group and the untreated group (panel A).

As mentioned above, the elimination half-life of cocaine is dependent on the actual dose. In this study, we first studied the PK profile of 60 mg/kg cocaine administered *via* intraperitoneal (i.p.) injection. According to our PK data depicted in Fig. 1A, 60 mg/kg cocaine had an elimination half-life of ~56 min and still showed a significant concentration of cocaine at four hours after the cocaine injection.

**Figure 2:**
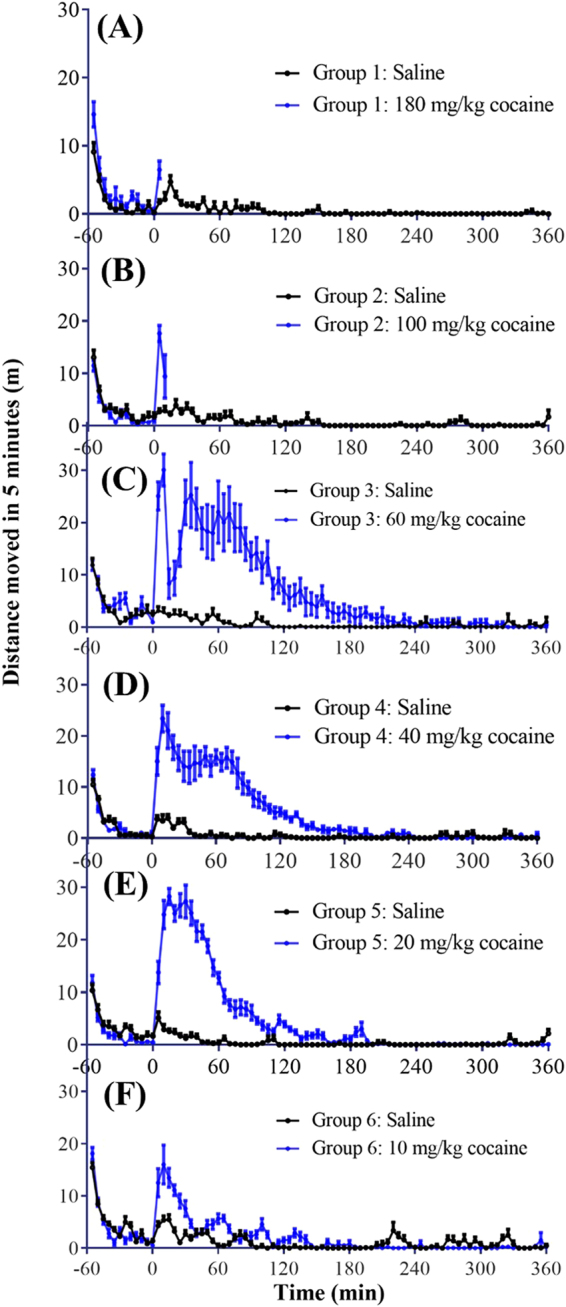
Cocaine-induced locomotor activity in rats. For each group of rats (n = 8), rats were first allowed to acclimate to the test chambers for 60 min before i.p. administration of cocaine or saline. Cocaine dose: (**A**) 180 mg/kg; (**B**) 100 mg/kg; (**C**) 60 mg/kg; (**D**) 40 mg/kg; (**E**) 20 mg/kg; (**F**) 10 mg/kg. Data are plotted as the mean ± s.e.m. meters traveled in 5-min bin during 7-hour tests. With the lethal doses in (**A**) to (**C**), the recording was terminated after the occurrence of death.

A significant blood concentration of cocaine is associated with various physiological/toxic effects. In rat models, it is convenient to observe cocaine-induced hyperactivity, convulsion (a symptom of seizure), and death. We carried out locomotor activity tests in rats using various i.p. doses of cocaine (10, 20, 40, 60, 100, and 180 mg/kg, n = 8 per dose). As shown in Fig. 2, changing the dose of cocaine from 10 to 60 mg/kg, the duration of cocaine-induced hyperactivity became longer and longer, roughly ~1 hour for 10 mg/kg, ~2 hours for 20 mg/kg, ~3 hours for 40 mg/kg, and ~4 hours for 60 mg/kg. The ~4-hours hyperactivity (Fig. 2C) induced by 60 mg/kg cocaine is consistent with the observed significant blood cocaine concentrations within the four hours after the cocaine administration.

### Correlation between blood concentration and lethality of cocaine

In addition to the hyperactivity, 60 mg/kg cocaine induced convulsion in six out of eight rats, and two rats died after convulsion (one at ~18 min and the other at ~24 min). The average time for the onset of cocaine-induced convulsion was ~10.3 min after administration. As shown in Fig. 1A, cocaine concentration in the blood reached a peak of ~7 μM (or ~7000 nM) at ~10 min after i.p. administration of 60 mg/kg cocaine. Comparison of the animal behavior data in Fig. 2C with the cocaine PK data in Fig. 1A suggests that ~7 μM is close to the threshold blood cocaine concentration leading to convulsion in rats. When the blood cocaine concentration is around 7 μM, the effects caused by cocaine in central nervous system could induce seizure, which manifests as convulsions in the behavior of rats. This is also a critical period, since persistent seizure is most likely to cause sudden death. Since rats would not have free movement during convulsion and/or after death, the observed average locomotion activity in Fig. 2C first reached a peak at ~10 min and then dropped sharply due to the occurrence of convulsions in several rats. Data from Fig. 1A also showed that the blood cocaine concentration maintained above 5 μM from 10 min to 60 min after cocaine administration. During this period, the behavioral effects of cocaine varied over a large range in rats, which is the reason for the big error bars in the figure when plotting the locomotion data. In some rats, a blood concentration in this range mainly produced hyperactivity. However, in the other rats, the blood concentration was high enough to maintain stereotyped behaviors, showing as fast repetitive head and/or foreleg movement when remaining in the corner of the test chamber^33^. After an hour, the blood cocaine concentration dropped gradually to a level below 5 μM. Majority of the rats recovered from the restricted component of cocaine’s effects and manifested hyperactivity as the behavioral effects of cocaine.

### Rescue effects of Albu-CocH1 and diazepam on the toxicity of a lethal dose (60 mg/kg, i.p.) of cocaine

**Figure 3:**
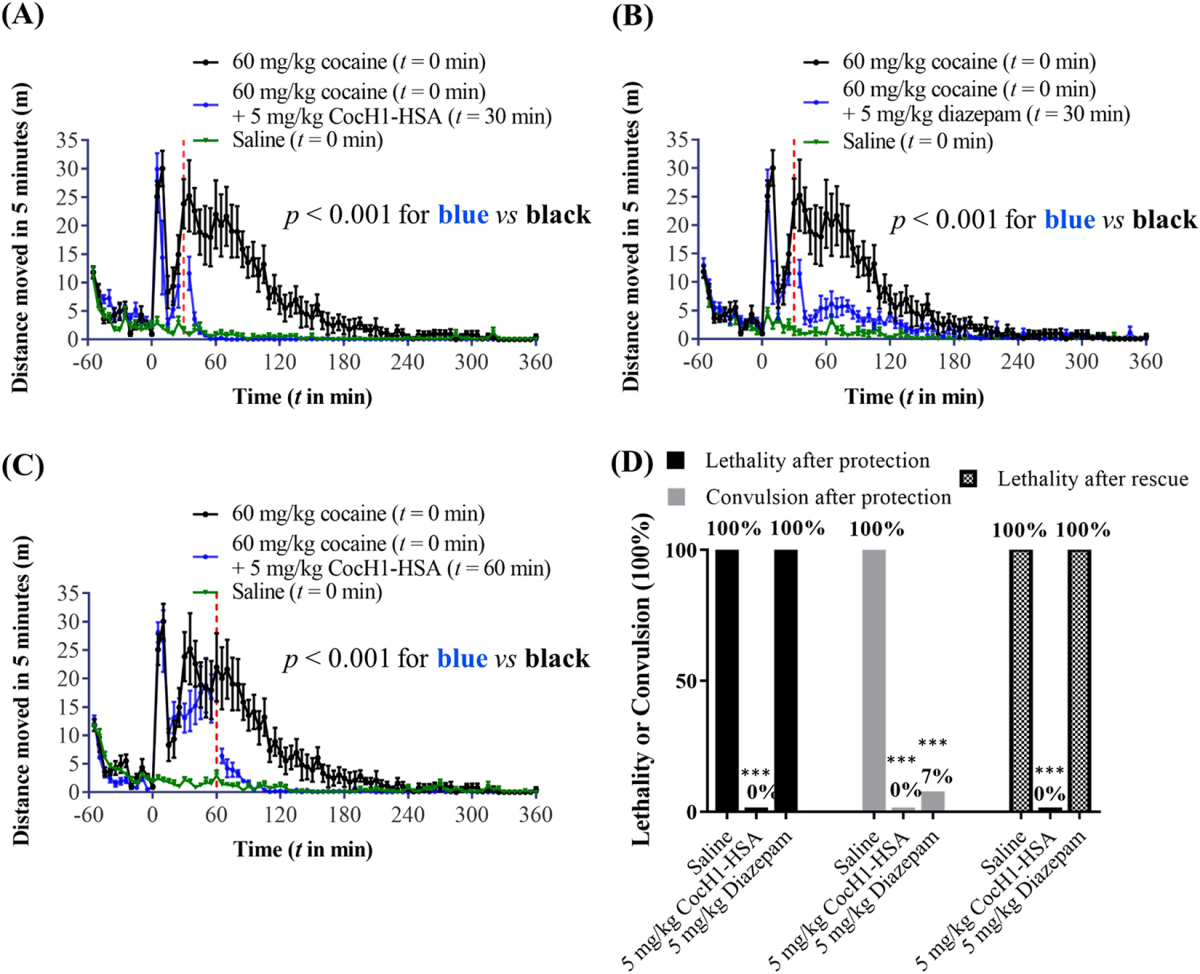
Effects of 5 mg/kg Albu-CocH1 and diazepam on cocaine-induced locomotor activity and toxicity in rats. (**A**–**C**) Rats (n = 8 per group) were allowed to acclimate to the test chambers for 60 min before i.p. administration of saline or 60 mg/kg cocaine. (**A**) 5 mg/kg Albu-CocH1 (i.v.) or (**B**) 5 mg/kg diazepam (i.p.) was administered at *t* = 30 min or (**C**) *t* = 60 min (Albu-CocH1 only) after the cocaine administration. Vertical red dashed lines indicate the time points when the interventions were introduced. Data are plotted as the mean ± s.e.m. meters traveled in 5-min bin during 7-hour tests. (**D**) Occurrence of convulsions and death in protection and rescue experiments. *p* < 0.001 (or ***) indicates the significant difference in the cocaine-induced hyperactivity/convulsion/lethality between the treated group and the corresponding untreated group.

Since the blood cocaine concentration reached the peak at ~30 min after i.p. administration of 60 mg/kg cocaine (Fig. 1A), in this set of experiments, we wanted to see whether administration of 5 mg/kg Albu-CocH1 or diazepam at 30 min after cocaine administration can help to reverse the cocaine toxicity, as shown in Fig. 3. As mentioned above, most of rats had convulsion after i.p. administration of 60 mg/kg cocaine, and about 25% rats died before 30 min after cocaine injection. Rescue procedures were only performed on rats that had convulsion but were still alive at 30 min (or 60 min) after cocaine administration.

The enzyme dose of 5 mg/kg used in our experiments is comparable to human dose of 300 mg per person, assuming that the body weight of an adult is around 60 kg. This dose has been proven safe for humans in the clinical trials for Albu-CocH1 which has an elimination half-life of ~8 hours in rats as shown in Fig. 1F and an elimination half-life of 43–77 hours in humans^10,11^. In addiction, 5 mg/kg is the up limit of diazepam dose range recommended for rat medication (http://ratguide.com/meds/central_nervous_system_drugs/diazepam.php). For the sake of comparison, diazepam was expected to be administered intravenously (i.v.) as the same as the administration route for Albu-CocH1. However, in our pilot study, 5 mg/kg diazepam administered i.v. caused strong sedative effects in control group (without cocaine administration) with all rats lost righting reflex and muscle tone immediately after injection and lasted for at least one hour. In the other group, rats received 5 mg/kg diazepam (i.p.) exhibited drowsiness with slow and deep respiration within 3 min. In addition, i.p. injection of diazepam was more commonly used in the other studies and has been approved to be able to prevent the cocaine-induced convulsion in the rats^34^. Therefore, we chose i.p. injection as the appropriate route to administer diazepam in this experiment.

According to data depicted in Fig. 3B, i.p. administration of 5 mg/kg diazepam significantly attenuated cocaine-induced hyperactivity. This behavioral result is largely attributed to the central inhibitory effects of diazepam, rather than acceleration of cocaine metabolism. On the contrary, according to data shown in Fig. 1C, diazepam actually slowed down elimination of cocaine and its metabolites (*p* = 0.049 for cocaine, *p* = 0.046 for benzoylecgonine, and *p* < 0.001 for norcocaine, according to statistical analysis using the two-way ANOVA). Due to the sedative effects of diazepam, rats generally had slower heart and breathing rates than normal during consciousness. This led to changed speed of hepatic blood flow, which could be the factor altering the clearance rate of cocaine in the rats.

In comparison, i.v. administration of 5 mg/kg Albu-CocH1 immediately and completely eliminated cocaine-induced convulsion and hyperactivity (Fig. 3A), which is consistent with the cocaine PK data depicted in Fig. 1B showing that cocaine was metabolized rapidly after the enzyme administration. In particular, blood cocaine concentration dropped from ~8 μM to ~0.06 μM in 2 minutes after enzyme administration.

Overall, Albu-CocH1 was able to completely and rapidly remove cocaine from the blood stream and relieve all of the physiological effects caused by cocaine. Therefore, Albu-CocH1 was much more effective than diazepam in cocaine toxicity treatment, if Albu-CocH1 or diazepam was given when the blood cocaine concentration reached the peak at 30 min after the 60 mg/kg cocaine administration.

In order to further study the effective window for Albu-CocH1 intervention, we tested the effects of 5 mg/kg Albu-CocH1 when Albu-CocH1 was administered (i.v.) at 60 min after the 60 mg/kg cocaine administration (Fig. 3C). Due to the individual differences discussed above, the average behavioral effects induced by 60 mg/kg cocaine (i.p.) in the first hour after administration differed from the control group. In fact, there were more rats in this treatment group showing prolonged stereotyped behaviors in the first hour after cocaine administration, resulting in relatively lower average locomotion activity shown in the figure. After i.v. administration of Albu-CocH1 at 60 min, all behavioral effects caused by cocaine, including elevated locomotion activity and stereotyped behaviors, were eliminated.

### Rescue effects of Albu-CocH1 and diazepam on the lethality of an extremely high dose (100 or 180 mg/kg, i.p.) of cocaine

As shown in Fig. 2A, increasing the lethal dose of cocaine from 60 mg/kg to 100 mg/kg, all rats started convulsion at ~6.3 min and died at ~18.1 min. This indicated that 100 mg/kg (i.p.) was LD_100_ for rats. Using 180 mg/kg cocaine, all rats had convulsion onset at ~2.8 min and died at ~4.1 min. Apparently, the higher the lethal dose of cocaine, the sooner the convulsion and death will occur. Hence, it would be a bigger challenge to detoxify subjects administered with further higher doses of cocaine in time.

Although the dose of 60 mg/kg examined above has already been sufficiently toxic, we would still like to further test the effectiveness of Albu-CocH1 and diazepam in protection and reuse rats from an even higher dose (180 mg/kg, LD_100_) of cocaine.

As shown in Fig. 1D, pretreatment of rats with 5 mg/kg Albu-CocH1 (i.v.) 1 min before i.p. administration of 180 mg/kg cocaine was able to rapidly and completely convert cocaine to EME, a physiologically inactive metabolite, once cocaine diffused into the blood. Blood cocaine concentrations (as well as the concentrations of cocaine metabolites including benzoylecgonine − the dominant metabolite shown in Fig. 1A in the absence of Albu-CocH1) at all time-points of blood sampling were negligible compared to the corresponding EME concentrations. As a result, in the presence of Albu-CocH1, 180 mg/kg cocaine did not induce convulsion or death in any rats tested, as shown in Fig. 3C and Table 1. The enzyme fully protected all of the rats from the acute toxicity of 180 mg/kg cocaine. For comparison, rats were injected with 5 mg/kg diazepam (i.p. or i.v.) 5 min (if i.p.) or 1 min (if i.v.) before i.p. administration of 180 mg/kg cocaine. Due to the anticonvulsant effects of diazepam, all rats (except one out of 14) did not show convulsion, but all rats died at ~9–10 min (Figure 3C and Table 1), later than the death of the control rats at ~4.1 min after cocaine administration. In conclusion, under the condition of 180 mg/kg cocaine, diazepam was only able to extend the time between the cocaine administration and death, without improving the survival.

**Table 1:** Effects of diazepam and CocH1-HSA on cocaine (180 mg/kg, i.p.) induced convulsion and lethality in rats.

Drugs used	Expt. method	Number of rats	Time after cocaine injection (min)		
			Convulsion	Death	Recovery^*f*^
Cocaine (180 mg/kg)	Control^*a*^	10	2.78 ± 1.03	4.07 ± 1.87	N.A.
Cocaine (180 mg/kg) and Diazepam (5 mg/kg)	Protection^*b*^	10 (i.p.)	N.O.	9.10 ± 2.11	N.A.
		4 (i.v.)	9.10 (one rat)	9.91 ± 5.55	N.A
	Rescue^*c*^	10 (i.p.)	2.75 ± 1.95	4.13 ± 2.76	N.A.
		4 (i.v.)	2.84 ± 1.91	4.14 ± 2.17	N.A.
Cocaine (180 mg/kg) and CocH1-HAS (5 mg/kg)	Protection^*d*^	10	N.O.	N.O.	N.A.
	Rescue^*e*^	10	2.82 ± 1.92	N.O.	4.2 ± 1.91

^*a*^Control experiment was performed by administration of 180 mg/kg cocaine (i.p.).

^*b*^Protection experiment was performed by pretreatment of rats with 5 mg/kg diazepam (i.p. for 10 rats and i.v. for 4 rats) 5 min (if i.p.) or 1 min (if i.v.) before administration of 180 mg/kg cocaine (i.p.).

^*c*^Rescue experiment was carried out by first administration of 180 mg/kg cocaine (i.p.). Then, in 1 min after the onset of cocaine-induced convulsion, rats were given 5 mg/kg diazepam (i.p. for 10 rats and i.v. for 4 rats).

^*d*^Protection experiment was performed by pretreatment of rats with 5 mg/kg CocH1-HSA (i.v.) 1 min before administration of 180 mg/kg cocaine (i.p.).

^*e*^Rescue experiment was carried out by first administration of 180 mg/kg cocaine (i.p.). Then, in 1 min after the onset of cocaine-induced convulsion, rats were given 5 mg/kg CocH1-HSA (i.v.).

^*f*^The clock time (starting from the cocaine administration) when the rats were fully recovered (occurrence of righting reflex and normal walk) from cocaine-induced convulsion.

In further rescue experiments, rats were given 180 mg/kg cocaine (i.p.) first, and then given 5 mg/kg Albu-CocH1 (i.v.) or diazepam (i.p. or i.v.) within 1 min after the onset of cocaine-induced convulsion. As shown in Fig. 3D and Table 1, diazepam did not rescue any rat; all rats died as if there was no diazepam administration. In comparison, the enzyme successfully rescued all rats; all rats survived and recovered (*i.e*. can have righting posture and move normally) at ~4.2 ± 1.9 min after the cocaine administration (or ~1 min after the enzyme administration). The rescue effects of the enzyme are consistent with cocaine PK data depicted in Fig. 1E showing the time course of blood cocaine concentration after the enzyme administration. As seen in Fig. 1E, immediately after the enzyme administration, cocaine in the blood was converted to physiologically inactive metabolite EME, which explains why all rats can recover so rapidly.

## Discussion

Cumulative evidence from animal tests and clinical studies have consistently demonstrated that the elimination half-life of cocaine is dependent on the cocaine dose in both rats and humans. The higher the dose, the longer the elimination half-life of cocaine. In addition, cocaine generally has a significantly shorter elimination half-life in rats compared to that in humans. Based on the animal data, whenever Albu-CocH1 is given to a living subject (no matter whether blood cocaine concentration has reached the peak or not), the remaining cocaine in the body will be converted rapidly to physiologically inactive EME and, thus, Albu-CocH1 can reverse the cocaine toxicity and help the subject to recover.

Further, according to our results, when the cocaine dose exceeds LD_100_, the time window for rescue is relatively narrow. However, the lower the cocaine dose, the wider the window for rescue. Our study has demonstrated that the rescue window associated with a lethal dose of 60 mg/kg (i.p.) is very reasonable. It should be noted that, for human with a 60 kg body weight, 60 mg/kg would be equivalent to 3.6 g, much higher than commonly used oral doses of 50–300 mg^35^; the minimum lethal dose of cocaine was estimated to be 1.2 g (http://drug.addictionblog.org/cocaine-overdose-how-much-amount-of-cocaine-to-overdose/). With a significantly lower dose of cocaine, the time window for rescue will be much wider, as in the cases of most cocaine overdose caused emergency department visits. In addition, 30 min for rats may correspond to hours for humans because rats can eliminate cocaine much more rapidly than humans.

It should also be noted that Albu-CocH1 is an investigational new drug (IND), known as TV-1380^10,11,36^, approved by the FDA for clinical trials in cocaine addiction treatment, rather than cocaine overdose treatment. The Phase I clinical trials have consistently proved that Albu-CocH1 (or TV-1380) in a single dose or repeated doses of 300 mg is safe for use in humans^10,11^. The Phase II clinical trial results “argue for development of improved enzymes with greater catalytic activity”^36^ in order to be effective with the desirable once-weekly dosing schedule for cocaine addiction treatment. The present study has demonstrated for the first time that Albu-CocH1 (or TV-1380) is likely more appropriate for cocaine overdose treatment, and all of the animal data discussed above consistently indicate that Albu-CocH1 is much more effective than diazepam in cocaine toxicity treatment. As Albu-CocH1 has been proven safe for use in humans, one may go ahead to conduct further clinical trials for the efficacy of CocH1 (or TV-1380) in cocaine overdose treatment.

In general, all of the animal data discussed above suggest that the key to cocaine toxicity treatment is to accelerate cocaine metabolism and rapidly convert cocaine to physiologically inactive metabolite EME. Once cocaine is completely converted to EME, the toxicity of cocaine will be reversed for the subjects. The general concept of enzyme therapy approach to cocaine overdose treatment may also be used to develop other drug-specific enzymes for effective treatment of the toxicity of other abused substances.

## Materials and Methods

### Materials

The purified Albu-CocH1 protein was prepared in our previous study^37^. Briefly, the Albu-CocH1 protein was expressed in stable CHO-S cells (developed in our lab using a lentivirus-based method) that can stably produce the Albu-CocH1 protein. The protein production was performed in an agitated bioreactor BioFlo/CelliGen 115 (Eppendorf, Hauppauge, NY). Albu-CocH1 in the culture medium was purified by using AlbuPure affinity chromatography on an ÄKTA Avant 150 system (GE Healthcare Life Sciences, Pittsburgh, PA). AlbuPure was ordered from ProMetic (Rockville, MD). The purified protein was dialyzed in a storage buffer (50 mM HPEPS, 20% Sorbitol, 1 M Glycine, pH 7.4) and stored at −80 °C before the use. (−)-Cocaine was provided by the National Institute on Drug Abuse (NIDA) Drug Supply Program (Bethesda, MD); and [^3^H](−)-Cocaine (50 Ci/mmol) was ordered from PerkinElmer (Waltham, Massachusetts). Diazepam (Valium®) injectable was from Hospira (Lake Forest, IL). All other chemicals were purchased from Thermo Fisher Scientific (Waltham, MA) or Sigma-Aldrich (St. Louis, MO).

### Animals

A total of one hundred and thirty seven (137) male Sprague-Dawley rats (220–250 g) were ordered from Harlan (Harlan, Indianapolis, IN), and housed initially as one or two rats per cage. All rats were allowed ad libitum access to food and water and maintained on a 12 h light/12 h dark cycle, with the lights on at 8:00 a.m. at a room temperature of 21–22 °C. Experiments were performed in a same colony room in accordance with the Guide for the Care and Use of Laboratory Animals as adopted and promulgated by the National Institutes of Health. The animal protocol was approved by the IACUC (Institutional Animal Care and Use Committee) at the University of Kentucky.

### Determination of pharmacokinetic profiles of Albu-CocH1 in rats

Rats were injected with the purified Albu-CocH1 protein *via* tail vein (5 mg/kg). Blood samples were taken from saphenous vein puncture using a needle. Approximately 40–75 µl blood was collected into a heparin-treated capillary tube at various time points after enzyme administration. Collected blood samples were centrifuged for 15 min at a speed of 5000 *g* to separate the plasma, which was kept at 4 °C before analysis. A sensitive radiometric assay^37^ using 100 μM (−)-cocaine was used to measure the enzyme concentration in plasma.

### Characterization of cocaine clearance in rats

Blood samples (40–75 μl) were collected from saphenous vein into a heparin-treated capillary tube at various time-points after the (−)-cocaine administration, and mixed immediately with 100 µl of 25 µM paraoxon (in 0.1% formic acid). Blood samples were stored at −80^o^C until analysis by using our previously developed LC-MS/MS method^38^ for simultaneously detecting the concentrations of (−)-cocaine and metabolites in blood samples.

### Locomotor activity assay

Cocaine-induced hyperactivity was monitored by using a video-tracking system in our lab. The locomotor activity tests were performed in high-density, non-porous plastic chambers measuring 50 cm (L) × 50 cm (W) × 38 cm (H) in a light- and sound-attenuating behavioral test enclosure (San Diego Instruments, San Diego, CA). Cumulative distance traveled was recorded by ANY-maze video tracking system (San Diego Instruments, San Diego, CA) to represent the locomotor activity. Before cocaine or saline administration, rats were allowed to acclimate to the test chambers for 1 h. The distance traveled was collected in 5-min bins. After cocaine or saline administration, rats were immediately returned to the test chamber for activity monitoring for 6 hours with or without administration of 5 mg/kg Albu-CocH1 or diazepam at 30 min or 60 min after the cocaine administration.

### Protection study in rats

Cocaine-induced acute toxicity was characterized in this study by the occurrence of convulsion and/or death. Cocaine-induced convulsion was defined as loss of righting posture for at least 5 seconds with the simultaneous presence of clonic limb movements^39^. Protection experiment was performed by pretreatment of rats with 5 mg/kg Albu-CocH1 (i.v.) or diazepam (i.p. or i.v.) 1 min (if i.v.) or 5 min (if i.p.) before administration of 180 mg/kg cocaine (i.p.). Following the cocaine administration, rats were immediately placed in containers for observation. The presence or absence of convulsion/death was recorded for 6 hours following cocaine administration^14^.

### Rescue experiment in rats

Rescue experiment was carried out by first administration of 60 or 180 mg/kg cocaine (i.p.). When the cocaine dose was 60 mg/kg, rats were given 5 mg/kg Albu-CocH1 (i.v.) or diazepam (i.p. or i.v.). When the cocaine dose was 180 mg/kg, rats were given 5 mg/kg CocH1-HSA (i.v.) or diazepam (i.p. or i.v.) within 1 min after the onset of cocaine-induced convulsion. Then, rats were immediately returned to the chambers or containers for observation.

### Statistical analysis

The Chi-squared contingency test was used to determine the overall significance of the incidence of convulsions and deaths against control group in protection and rescue experiments. The two-way repeated measures analysis of variance (ANOVA) was used to determine the significance of treatment effect on the cocaine induced locomotion activity. Cocaine PK data were analyzed by using two-way analysis of variance (ANOVA) method and *post hoc* Dunnett’s test. All of the statistical analyses were carried out using the SigmaPlot software (Systat Software, San Jose, CA).
